# Exploring the sports participation, muscle-strengthening exercise and active commuting with comorbidity of depression and anxiety among Chinese children and adolescents: a cross-sectional study

**DOI:** 10.3389/fpsyg.2024.1338190

**Published:** 2024-08-27

**Authors:** Bin Feng, Fuchen Luo, Yu Chen, Yuhang Zhao, Ping Wang, Ran Bao

**Affiliations:** ^1^Department of Physical Education, Tianjin College, University of Science and Technology Beijing, Beijing, China; ^2^Department of Physical Education, Beijing Zhengze School, Beijing, China; ^3^Institute of Sports Science, Harbin Sport University, Harbin, China; ^4^Department of History, Fudan University, Shanghai, China; ^5^Centre for Active Living and Learning, University of Newcastle, Callaghan, NSW, Australia; ^6^College of Human and Social Futures, University of Newcastle, Callaghan, NSW, Australia

**Keywords:** sport participation, muscle-strengthening exercise, active school travel, depression, adolescents, children

## Abstract

Prior research has shown that physical activity (PA) is a crucial element for preserving and enhancing health, particularly among children and adolescents, and consistent engagement in PA offers numerous advantages for sustaining typical physical and mental well-being.

**Purpose:**

Hence, the primary objective of this study was to examine the relationship between sport participation, muscle-strengthening exercise (MSE), and active commuting (AC) in the co-occurrence of depression and anxiety in Chinese children and adolescents.

**Method:**

This cross-sectional investigation occurred in various cities across the southeastern region of China between March 2021 and October 2021. A convenient sampling method was utilized. We invited children and adolescents to participate in the questionnaire survey. A total of 1,996 participants completed the questionnaires with the endorsement of their parents or guardians under the supervision of schoolteachers and headmasters. Girls comprised 47.5% of the participants, and the average age of participants was 14.8 ± 2.0 years. We conducted a logistic regression analysis, including 95% confidence intervals, to explore the association between sports participation, MSE, AC, and the co-occurrence of depression and anxiety.

**Results:**

No significant association was observed between weekday active commuting for travelling to and from school and MSE and the comorbidity of depression and anxiety in children and adolescents. A negative association was only detected for those who engaged in muscle-strengthening exercises 4 days a week (OR = 0.540, 95% CI = 0.345–0.845) compared to those who did not partake in such exercises.

**Conclusion:**

The present study has provided evidence of the connection between sports participation and the co-occurrence of depression and anxiety among Chinese children and adolescents. Sports participation is more likely to help adolescents relieve anxiety and depression than AC, MSE. In forthcoming research, it is imperative to delve deeper into strategies that enhance the impact of sports on the mental well-being of children and young individuals. Furthermore, optimizing the magnitude of this effect may be achievable by focusing on neurobiological, behavioral, and psychosocial mechanisms.

## 1 Introduction

Mental health is closely related to adolescents’ learning and development, interpersonal relationships, self-esteem, and self-awareness (Demirci et al., 2022; Liu T. et al., 2023). Mental health refers to a person’s regular, stable, and adaptive state in thought, emotion, and behavior, so a healthy mental state can help children and adolescents better grasp learning content and face life’s challenges (Sonuga-Barke et al., 2016). Shanahan and other scholars (2014) (Shanahan et al., 2011) found that the effect of mental health on children’s school performance was significant, with students in better mental health performing better academically than those in worse mental health. In addition, Collishaw (2015) points out that mental disorders may lead to impaired social functioning in adolescents, which in turn may affect their relationships. However, many adolescents can face mental health issues such as anxiety, depression, and attention-deficit/hyperactivity disorder, which can lead to emotional and behavioral problems and even affect their learning and development (Sonuga-Barke et al., 2016). Depression and anxiety often co-exist, and the causes and risk levels are similar and related. For example, genetic factors and stress from life events all play a role in the onset of depression and anxiety and are all influenced by personality traits and social factors (Panza et al., 2020). In addition, depression and anxiety also share many clinical features and diagnostic tools, such as the presentation of anxiety and depression symptoms is similar (Eijsbouts et al., 2021), and commonly used assessment tools such as the Hamilton Depression Scale and the Baker Self-Rating Anxiety Scale (Zhou et al., 2022a). Therefore, in terms of treatment methods and interventions, the management of depression and anxiety can learn from each other, such as cognitive behavioral therapy, medication, and psychological counselling. Wakschlag et al. (2019) study found that the treatment effect of early intervention in children and adolescents is better, which can effectively reduce the occurrence of psychological disorders. At the same time, early intervention and treatment can prevent the long-term impact of mental health problems in children and adolescents on their lives and work as adults (Yoshikawa et al., 2012). Starting from the importance of mental health, Hermann et al. (2022) analyze that mental disorders can negatively affect a person’s thoughts, feelings, and behavior in many ways, especially considering the attention-seeking and emotionally sensitive characteristics of adolescents. The incidence of mental disorders among children and adolescents has been increasing year by year, which means that schools and families need to pay more attention to mental health problems and take action to prevent and treat them. By providing services such as mental health education and psychological counselling, adolescents can be helped to better understand and cope with their emotional and behavioral problems, allowing them to build confidence, healthy relationships, and self-perception (Coghill and Sonuga-Barke, 2012).

Physical activity (PA) is an essential part of maintaining and promoting health (Pinto et al., 2020; Shen et al., 2020; Chen Z. G. et al., 2022; Fan et al., 2022; Li et al., 2024), especially for children and teenagers, and regular PA has many benefits not only in maintaining normal functioning physically and mentally (Lindsay-Smith et al., 2019; Liu et al., 2022; Li et al., 2023; Zheng et al., 2023) but also in academic ability and social communication (Liu et al., 2022; Zhang et al., 2023). Physically, PA directly promotes cardiovascular health, continues human vitality, and reduces the risk of obesity by building bone and muscle strength (Luan et al., 2019; Zheng et al., 2023). Studies have also shown that PA is negatively correlated with the incidence of chronic diseases such as diabetes and cardiovascular disease (Sallis et al., 2021). Mentally, regular PA can relieve mental stress and reduce the incidence of depression and anxiety symptoms in adolescents (Kang et al., 2021; Wolf et al., 2021; Zheng et al., 2023). More importantly, the team sports attached to PA can boost self-confidence, which is beneficial for fostering a sense of cooperation and social inclusion (Yan et al., 2024b). Cognitively, appropriate regular PA can relieve mental stress and reduce the incidence of depression and anxiety symptoms in children and adolescents. It helps improve attention and memory (Zhou et al., 2022b) and promotes academic performance (Li et al., 2023). The World Health Organization (WHO) prioritises PA in its guidelines, recommending that children and adolescents get at least 60 min of moderate-to-vigorous physical activity (MVPA) per day (Yan et al., 2024b). Regular PA can play a crucial role in promoting the overall development of young people and fostering healthy lifelong habits.

Regarding sports participation, it is the actions and attitudes of students initiatively participating in sports activities, which is just an overall description of sports objectives (Ren et al., 2021; Santos-Pastor et al., 2022; Liu T. et al., 2023). From the angle of sociology and psychology, sports participation is a significant way to realize kids’ socialization, which refers to a socialization procedure in essence (Dorsch et al., 2009; Yan et al., 2023). Apart from that, sports participation indicates success and the investment of psychological energies, and different students devote different energies to activities according to time and objectives (Farias et al., 2017). Such a concept includes the activity’s external indicators (e.g., mood) and the exercise goals. As pointed out by Wicker and Frick (2015), females and males exercising more frequently have better health. However, for females, the difference in subjective health is partly attributed to education, deprivation of economy, and family-work pressure. As Eime et al. (2013) claimed, regular physical activities prevent chronic diseases and decrease the risks of premature death. Likewise, Kantomaa et al. (2015) demonstrated that high physical activity levels have a positive relationship with the health of adolescents. As suggested by other research, exercise is favorable to enhancing people’s self-rated health (Marques et al., 2017; Shi C. et al., 2022).

Sports participation is closely linked to mental well-being. Research indicates that consistent physical activity can have favourable effects on various facets of psychological health, including reducing depressive symptoms, alleviating mood, and enhancing self-esteem (Appelqvist-Schmidlechner et al., 2018). Engaging in sports facilitates the release of endorphins and natural mood enhancers and offers a channel for relieving pressure (Parker et al., 2014). Moreover, physical activity has the potential to enhance the quality of sleep, a factor of significant importance for maintaining good psychological health (Wickham et al., 2020). Furthermore, participating in physical activities can provide social support, a crucial factor in promoting psychological well-being (Laird et al., 2018; Zheng et al., 2023). Exercise can also give a sense of purpose by serving as a distraction from negative thoughts (Eime et al., 2013). Therefore, Easterlin et al. (2019) emphasized the significance of integrating sports participation into daily life to maintain psychological health. Simultaneously, it is crucial to identify enjoyable and sustainable physical activities to ensure ongoing involvement and psychological well-being.

Participating in adequate and consistent PA is strongly linked to higher levels of health-related physical fitness in children and adolescents, making it a significant contributor to improved physical fitness (Shi J. et al., 2022), as demonstrated in the literature. However, these studies primarily examined overall physical activity levels rather than focusing on the specific modes. Consequently, recent research has emphasized the need to investigate the relationships between different modes of physical activity and health-related physical fitness among children and youth. Children and adolescents engage in various modes of physical activity, some of which have demonstrated significant associations with health outcomes and fitness components. Among these different modes, sports participation, muscle-strengthening exercise (MSE), and active school travel (AST) stand out as three fundamental forms of physical activity among adolescents (Huang et al., 2021; Shi J. et al., 2022). Sports participation often occurs within school settings, while AST is a daily routine, and both modalities play pivotal roles in monitoring an active lifestyle on a global scale (Nweke et al., 2019). Concerning muscle-strengthening exercise (MSE), the WHO recommends that young individuals engage in such activities a minimum of three times per week (Chaput et al., 2020). Moreover, a considerable amount of evidence indicates a connection between sports participation, muscle-strengthening exercise (MSE), and active school travel (AST) with mental health outcomes in adolescents (Lee et al., 2019; Huang et al., 2021; Shakespear-Druery et al., 2021; Müller et al., 2022).

The close connection between long-term physical activity and mental disorders, such as depression and anxiety, can be attributed to alterations in brain function and structure (Matta Mello Portugal et al., 2013). Specifically, long-term physical activity participation can promote neurogenesis, angiogenesis and synaptogenesis, potentially mitigating depression and anxiety (Matta Mello Portugal et al., 2013). Moreover, sports participation necessitates concentration, emotional regulation, and coping strategies (Robazza et al., 2004). These elements, associated with enhanced physical performance, may alleviate depression and anxiety among participants. Additionally, the central nervous system, accountable for neuromuscular activities and task execution, may offer anticipatory control that could help ward off psychological depression and anxiety (Matta Mello Portugal et al., 2013). Further, the underlying mechanisms connecting muscular training activities and mental disorders, such as depression and anxiety, remain to be fully understood. Potential explanations could include enhanced social interaction and expectations (Gordon et al., 2017). However, there is a shortage of evidence on the benefits of sports participation, MSE, and AC about the comorbidity of depression and anxiety in adolescents. To address this research gap, the current study aims to explore the relationship between sports participation, MSE, and AC and the coexistence of depression and anxiety among Chinese adolescents. This investigation intends to provide empirical evidence to fill this gap in the existing literature.

## 2 Methods

### 2.1 Participants and procedure

The cross-sectional study employed a convenient sampling method. The data collection was conducted in the Southeast regions of China between March 2021 and October 2021. The current study included public school-school adolescents in grades 4, 5, 7, 8, 10, and 11. Students in grades 9 and 12 were excluded as they prepared for the General High School Academic Proficiency Test and the National College Entrance Examination, respectively. For each grade, two classes were selected using a convenient sampling method. Before the commencement of data collection, participants were provided with detailed instructions on the data collection process. Children and adolescents with physical or intellectual disabilities were not included in the current study. A total of 2,374 participants consented to participate in this survey. After removing invalid data (such as providing invalid answers or missing answers), 1996 children and adolescents who completed the study and offered valid information were included in the final analysis. Girls comprised 47.5% of the participants, and the average age of participants was 14.8 ± 2.0 years. Formal written permission and consent were obtained from participants and their parents. Participants who provided information on the relevant variables were included in the study. Those who did not report data on required variables (e.g., independent variables, outcomes, and covariates) were excluded from the initial sample. Additionally, all respondents and their guardians or parents were informed that participation in the survey was entirely voluntary. This study was approved by the Research Board at Shanghai University of Sport (Approval Number: 102772021RT071).

### 2.2 Measures

#### 2.2.1 Sport participation

A single item was used to analyze sports participation, stated below: “Over the past 12 months, have you participated in a sports club, a sports team, or a sport-related activity?” The possible answers to such a question include never, 1–3 times per month, 1–2 times per week, and above three times per week. Such a measurement item has been confirmed to be a reliable and effective question for evaluating adolescents’ sports participation (Lian et al., 2021; Liu T. et al., 2023).

MSE was evaluated through the inquiry: *“Over the past week, how frequently did you participate in activities aimed at strengthening or toning your muscles, such as push-ups, sit-ups, or weightlifting?”* The possible responses were: *0 = none, 1 = 1 day, 2 = 2 days, 3 = 3 days, 4 = 4 days, 5 = 5 days, 6 = 6 days, and 7 = 7 days.* This metric has been established as a reliable and valid tool for evaluating MSE in Chinese children and adolescents (Xin et al., 2021; Wang et al., 2024). According to the World Health Organization’s guidelines, individuals who reported engaging in such activities for three or more days were categorised as meeting the MSE recommendation, while those who reported fewer than 3 days were classified as not meeting the guideline (Chaput et al., 2020).

AST was measured by using two distinct inquiries: (1) *“During weekdays, how frequently did you use active means such as walking or cycling to travel to school?”* and (2) *“On weekdays, how often did you use active transportation methods like walking or cycling to return home after school?”* For both questions, respondents selected from 0 to 5 days as their response. These two items have been used in Chinese children and adolescents (Gu and Chen, 2020; Chen et al., 2021).

#### 2.2.2 Depressive symptoms and anxiety

The Chinese version of the 9-item Patient Health Questionnaire (PHQ-9) was applied to analyze depressive symptoms. This tool included nine items related to depressive symptoms within the past 2 weeks. Each item used a Likert four-point scale, from 0 (none) to 3(almost daily). The total score ranged from 0 to 27. The higher the score, the more severe depressive symptoms will be. According to PHQ-9 scoring, the severity of depressive symptoms was classified as 0-4(minimal), 5-9(mild), 10-14(moderate), 15-19(moderately severe), and 20-27(severe). The psychological measurement properties of PHQ-9 have been tested on Chinese children, showing adequate reliability and validity (Wang et al., 2014; Chen J. K. et al., 2022).

7-item generalized Anxiety Disorder Scale (GAD-7) was used to assess anxiety disorders. This scale was composed of 7 items within the past 2 weeks. The answer to each item is applied to a Likert four-point scale (from 0 to 3). The total score of GAD-7 ranged from 0 to 21. The higher the score, the more severe the degree of anxiety. The severity of anxiety could be divided into four categories, including minimal (0–4), mild (5–9), moderate (10–14) and severe (15–21). The translated GAD-7 was widely applied among Chinese children and adolescents, showing acceptable reliability and validity (Sun et al., 2021; Zhang et al., 2021).

### 2.3 Statistical analysis

SPSS 26.0 was employed for all statistical analyses. As the proportion of missing data was less than 5%, the missing data was deleted from the analysis. Descriptive statistics, including the percentage for categorical variables and the mean with standard deviation for continuous variables, were utilized to present relevant features. In further analysis, the following variables were used as concomitant variables, including gender, grade, age, place of residence, rich degree of family, and whether living with parents or not (Appelqvist-Schmidlechner et al., 2018; Easterlin et al., 2019; Wickham et al., 2020). Partial correlation was adopted to investigate associations among sports participation, muscle-strengthening exercise (MSE), AST, depression, and anxiety while accounting for sociodemographic factors, BMI, gender, and grade. Generalized Linear Models with Ordinal Logistic Regression (OR) were applied to assess the relationships between sports participation, muscle-strengthening exercises, and active commuting with depressive symptoms and anxiety disorders. This evaluation was conducted after adjusting for all previously mentioned covariates. The statistical significance threshold was set at *p* < 0.05.

## 3 Results

Table 1 shows the characteristics of the sample. A total of 1,996 participants were included in the final analysis. Specifically, 47.5% were girls, with a mean age of 14.8 ± 2.0 years and a mean BMI of 20.8 ± 5.0. Most participants (67.6%) resided in urban areas, while 21.4% lived in suburban areas and 10.9% in rural areas. Half of the participants had siblings, and the majority (84.6%) lived with their parents. Over half of the students reported that their fathers had only a high school diploma or less, while 36.8% of fathers had an undergraduate degree and 6.4% had a graduate degree. Similarly, over half of the students indicated that their mothers had only a high school diploma or less, with 35.2% having an undergraduate degree and only 3.5% having a graduate degree.

**Table 1:** Sample characteristics.

		*n*/mean	%/sd
Continuous variables			
Age		14.08	±2.0
Body mass index		20.81	±5.0
Categorical variables			
Gender			
	boy	1,048	52.5
	Girl	948	47.5
	Total	1996	100.0
Residence			
	Rural	218	10.9
	Suburban	428	21.4
	Urban	1,350	67.6
	Total	1996	100.0
Siblings			
	Yes	1,005	50.4
	No	991	49.6
	Total	1996	100.0
Live with parent			
	Yes	1,689	84.6
	NO	307	15.4
	Total	1996	100.0
Grade			
	4	218	10.9
	5	242	12.1
	7	295	14.8
	8	356	17.8
	10	484	24.2
	11	401	20.1
	Total	1996	100.0
Mother Education Level			
	Middle school or below	576	28.9
	High school	558	28.0
	Undergraduate	735	36.8
	Graduate	127	6.4
	Total	1996	100.0
Father Education Level			
	Middle school or below	707	35.4
	High school	481	24.1
	Undergraduate	703	35.2
	Graduate	105	5.3
	Total	1996	100.0
Sport participation			
	Never	1,190	59.6
	1--3 times per month	312	15.6
	1--2 times per week	350	17.5
	3 or more times per week	144	7.2
	Total	1996	100.0
AST in weekday (go to school) 1			
	0	917	45.9
	1 day	104	5.2
	2 day	109	5.5
	3 day	71	3.6
	4 day	64	3.2
	5 day	731	36.6
	Total	1996	100.0
AST in weekday (after school) 2			
	0	828	41.5
	1 day	115	5.8
	2 day	108	5.4
	3 day	80	4.0
	4 day	53	2.7
	5 day	812	40.7
	Total	1996	100.0
Muscle strengtheninghoning exercises (past 7 days)			
	0	726	36.4
	1 day	370	18.5
	2 day	420	21.0
	3 day	228	11.4
	4 day	87	4.4
	5 day	165	8.3
	Total	1996	100.0
Depressive			
	No	1,671	83.7
	Yes	325	16.3
	Total	1996	100.0
Anxiety			
	No	1753	87.8
	Yes	243	12.2
	Total	1996	100.0

Regarding sports participation, 15.6% of students engaged 1–3 times weekly, 17.5% for 1–2 times, and 7.2% for three or more times. For muscle-strengthening exercises, 36.4% abstained, 18.5% engaged once weekly, 21.0% for twice, 11.4% for three times, 4.4% for four times, and 8.3% for five times. Additionally, 36.6% actively commuted to school daily, with 3.2, 3.6, 5.5, and 5.2% doing so for four, three, two, and 1 day(s), respectively. Similarly, 45.9% actively commuted home, with 2.7, 4.0, 5.4, and 5.8% doing so for four, three, two, and 1 day(s), respectively. Furthermore, 16.3% of respondents reported symptoms of depression, while 83.7% did not. Similarly, 12.2% reported symptoms of anxiety, while 87.8% did not. Concerning the co-occurrence of depression and anxiety, 9.9% of respondents reported experiencing both, 8.7% reported at least one, and 71.4% reported neither.

Table 2 outlines the correlation coefficients between demographic variables, sports participation, and depression and anxiety symptoms. Significant correlations were found between muscle-strengthening exercises and depression symptoms (*r* = −0.066, *p* = 0.003) and comorbidity (*r* = −0.045, *p* = 0.043). Furthermore, no significant correlation was found between sports participation, active commuting travel, depression symptoms, anxiety symptoms, or comorbidity in children and adolescents.

**Table 2:** The correlation coefficients between demographic variables, sports participation and depression and anxiety symptoms.

Variables	Age	BMI	Gender	Grade	Residence	Siblings	Live with parent	Mother education level	Father education level	AST in weekday (go to school)	AST in weekday (after school)	Muscle strengthening exercises (past 7 days)	Sport participation	Depressive	Anxiety	Comorbidity
1.Age	1															
2.BMI	0.190**	1														
Gender	0.01	−0.093**	1													
Grade	0.958**	0.188**	0.02	1												
Residence	−0.01	0.03	0.01	−0.02	1											
Siblings	−0.054*	−0.051*	0.04	−0.01	−0.310**	1										
Live with parent	0.02	0.03	−0.01	0.02	−0.137**	0.077**	1									
Mother Education Level	−0.00	−0.04	0.057*	−0.04	0.412**	−0.424**	−0.160**	1								
Father Education Level	−0.02	−0.03	0.04	−0.063**	0.397**	−0.430**	−0.153**	0.730**	1							
AST in weekday (go to school)	−0.064**	−0.04	−0.069**	−0.077**	0.00	−0.02	0.03	0.072**	0.066**	1						
AST in weekday (after school)	−0.085**	−0.03	−0.048*	−0.106**	0.060**	−0.069**	0.01	0.109**	0.122**	0.824**	1					
Muscle strengthening exercises(past 7 days)	−0.182**	−0.085**	−0.132**	−0.196**	0.158**	−0.094**	−0.052*	0.176**	0.143**	0.088**	0.091**	1				
Sport participation	−0.237**	−0.085**	−0.107**	−0.259**	0.122**	−0.132**	−0.053*	0.174**	0.192**	0.131**	0.131**	0.362**	1			
Depressive	0.083**	0.070**	−0.00	0.085**	0.04	−0.00	0.102**	0.02	0.04	0.01	0.01	−0.066**	−0.01	1		
Anxiety	0.059**	0.04	0.04	0.063**	0.04	−0.01	0.071**	0.03	0.059**	0.01	0.00	−0.01	−0.01	0.653**	1	
Comorbidity	0.079**	0.063**	0.01	0.082**	0.04	−0.01	0.096**	0.03	0.054*	0.01	0.00	−0.045*	−0.01	0.920**	0.897**	1

**p ≤ 0.01, *p ≤ 0.05.

The relationship between sports participation, muscle strengthening, active commuting, and comorbidity is displayed in Table 3. When compared to individuals who never participated in sports, participating in sports 1–3 times per week (OR = 1.023, 95%CI = 0.723 to 1.447, *p* = 0.899), 1–2 times per week (OR = 1.175, 95%CI = 0.832 to 1.660, *p* = 0.360), and more than three times per week (OR = 0.791, 95%CI = 0.453 to 1.380, *p* = 0.409) was not associated with a lower incidence of comorbidity. Additionally, no association between active commuting to school and back home on weekdays and comorbidity in adolescents was found. A negative association was only found for participating for 4 days (OR = 0.540, 95% CI = 0.345–0.845) and comorbidity compared to those who did not participate in muscle strengthening exercise.

**Table 3:** Parameter estimates obtained from the logistic regression model based on the outcome of AST, MSE, and sport participation.

Parameter			95% Wald confidence interval		Hypothesis test				95% Wald confidence interval for exp (B)	
Threshold		B	Lower	Upper	Wald chi-square	df	Sig.	Exp (B)	Lower	Upper
[AST in weekday (go to school) = 6]	[Comorbidity = 0]	0.02	−2.63	2.67	0.00	1	0.99	1.02	0.07	14.50
	[Comorbidity = 1]	0.79	−1.86	3.45	0.34	1	0.56	2.21	0.16	31.42
[AST in weekday (go to school) = 5]		0.02	−0.45	0.50	0.01	1	0.93	1.02	0.64	1.64
[AST in weekday (go to school) = 4]		0.29	−0.59	1.17	0.42	1	0.52	1.34	0.56	3.22
[AST in weekday (go to school) = 3]		0.38	−0.35	1.10	1.03	1	0.31	1.46	0.70	3.01
[AST in weekday (go to school) = 2]		0.03	−0.61	0.67	0.00	1	0.92	1.03	0.54	1.95
[AST in weekday (go to school) = 1]		−0.30	−0.97	0.36	0.81	1	0.37	0.72	0.38	1.44
[AST in weekday (after school) = 6]		0^a^								
[AST in weekday (after school) = 5]		−0.05	−0.53	0.42	0.05	1	0.83	0.95	0.59	1.53
[AST in weekday (after school) = 4]		−0.56	−1.52	0.40	1.30	1	0.25	0.57	0.22	1.49
[AST in weekday (after school) = 3]		0.39	−0.27	1.05	1.34	1	0.25	1.48	0.76	2.85
[AST in weekday (after school) = 2]		0.05	−0.60	0.69	0.02	1	0.89	1.05	0.55	2.00
[AST in weekday (after school) = 1]		0.01	−0.60	0.62	0.00	1	0.98	1.01	0.55	1.85
[Muscle strengtheninghoning exercises(past 7 days) = 6]		0^a^								
[Muscle strengtheninghoning exercises(past 7 days) = 5]		−0.48	−1.01	0.05	3.14	1	0.08	0.62	0.36	1.05
[Muscle strengtheninghoning exercises(past 7 days) = 4]		−0.62	−1.32	0.08	3.00	1	0.08	0.54	0.27	1.09
[Muscle strengtheninghoning exercises(past 7 days) = 3]		−0.62	−1.06	−0.17	7.26	1	0.01	0.54	0.34	0.85
[Muscle strengtheninghoning exercises(past 7 days) = 2]		−0.28	−0.60	0.05	2.76	1	0.10	0.76	0.55	1.05
[Muscle strengtheninghoning exercises(past 7 days) = 1]		−0.28	−0.62	0.06	2.69	1	0.10	0.75	0.54	1.0
[Sport participation = 4]		0^a^								
[Sport participation = 3]		−0.23	−0.79	0.32	0.68	1	0.41	0.79	0.45	1.38
[Sport participation = 2]		0.16	−0.18	0.51	0.84	1	0.36	1.17	0.83	1.66
[Sport participation = 1]		0.02	−0.32	0.37	0.02	1	0.90	1.02	0.72	1.45

Dependent Variable: Comorbidity, Model: (Threshold), whether the MVPA guidelines are met, whether the screen guidelines are met, whether the sleep guidelines are met, AST in weekday (go to school), AST in weekday (after school), Muscle-strengthening exercises (past 7 days), Sport participation. ^a^ Set to zero because this parameter is redundant.

## 4 Discussion

This study aimed to investigate the correlation between sports participation, MSE, and AC and the concurrent occurrence of depression and anxiety in children and youth. The findings indicated that there was no significant association between weekday active commuting for school travel and MSE with the coexistence of depression and anxiety in children and adolescents. Notably, a negative association was observed only for those who participated in muscle-strengthening exercises 4 days a week compared to those who did not engage in such exercises.

In China, studies reveal significant levels of depression and anxiety among children and adolescents. For example, in Shanghai, middle school surveys found depression rates around 15.7% and anxiety rates at 23.9% (Jörns-Presentati et al., 2021). Urban areas often show higher prevalence due to academic pressure and family expectations, while rural areas face challenges in accessing mental health services (Murry et al., 2011). Gender differences exist, with females typically reporting more symptoms (Salk et al., 2017).

Our findings affirm the idea that sports participation is associated with a reduced propensity for depression and anxiety in children and adolescents. This is consistent with the conclusions of recent research firstly from the physiological benefits of sports participation, the frequency of physical exercise participation, and the release of endorphins in a positive light, more and faster production of “feel-good” hormones can improve mood and promote well-being (Leahy et al., 2020). In addition, participation in sports is more effective in transferring negative psychology, which is particularly important for sensitive students because they are often influenced by the surrounding environment and are under tremendous psychological pressure (Salim and Winter, 2022; Pachankis and Jackson, 2023). According to research, in addition to genetic factors, the psychological problems of primary and secondary school students at this stage are mainly caused by factors such as learning pressure, family conditions, peer communication, and so on (Fegert et al., 2020; Liu X. Q. et al., 2023). Psychological problems often manifest through emotions and behaviors, such as lack of sleep, anxiety, depression, loneliness, confrontation, inattention, etc. (Hen et al., 2022). Therefore, from the efficiency perspective, sports’ role in maintaining and improving people’s mental health is unique and even irreplaceable. Because of these reasons and manifestations, physical education teachers should take the initiative based on the characteristics of young people and actively participate in promoting and improving the mental health of young people.

In addition, the social dimension intertwined with sports can also suppress some negative emotions. Children and adolescents who participate in sports tend to do so in teams, which can foster their sense of belonging and enhance their self-esteem (Martin et al., 2018). This sense of connection and worth can significantly reduce feelings of loneliness and loneliness, and being isolated is thought to be a significant trigger for depression and anxiety (Dale et al., 2019). At a time when adolescents must face the combined challenges of identity issues and peer pressure, engaging in physical activity can help distract them from these pressures and reduce the likelihood of developing depression (Howie et al., 2020; van Sluijs et al., 2021). Therefore, the school sports activities should be oriented to all students, focusing on collective and team activities and giving priority to diversified sports such as basketball, football, and volleyball (Carter-Thuillier et al., 2023; Eluère et al., 2023). This can ensure that everyone participates and has special skills, nourishing young people’s body and mind with various sports activities. It can be seen that compared with other activities, sports activities pay more attention to the rules of games and teamwork to cultivate students’ communication skills and interpersonal relationship-handling methods (Yu and Jiang, 2017).

Participation in sports can also build resilience and coping skills in adolescents, and grit can play a role in resisting negative emotions. In sports, young people often face situations that force them out of their “comfort zone, “a continuous opportunity to challenge their inertia, physical strength, and motor skills (Huang et al., 2021) through years of perseverance in honing the will to finally enjoy the process of happiness, eventually developing perseverance and optimistic character. The challenges and setbacks encountered in athletic pursuits can, therefore, teach adolescents to manage adversity and stress more effectively, thus making them less susceptible to depression.

Although participation in physical activity is protective against depression in adolescents, it is important to recognize that it is not the only solution. The influence of family environment, academic pressure, and other factors on adolescents’ mental health cannot be ignored. Therefore, to promote mental health and prevent mental health problems, we must start with many aspects and comprehensive treatment. For schools, it is necessary to strengthen monitoring and precise intervention. Physical education teachers should make full use of the characteristics of physical education disciplines, give play to the advantages of strong interpersonal skills of physical education teachers, and form a helping relationship with teenagers with psychological imbalances to prevent sudden psychological problems (Yan et al., 2023; Yim et al., 2023; Yan et al., 2024a,b). Although evidence suggests that promoting youth participation in physical activity is an effective strategy to reduce the incidence of depression and anxiety disorders, the specific types and amounts of physical activity that are most beneficial for the mental health of adolescents with different personalities still need to be refined. More advice is needed on how to most effectively promote these activities among children and adolescents (Guinto et al., 2022).

In contrast to earlier research findings (Larouche et al., 2014), the current study observed no correlation between active commuting and self-reported depression and anxiety. Given that active commuting is linked to increased physical activity levels, it is plausible that it could contribute to a reduction in depression and anxiety among children and adolescents. Prior investigations indicated a positive correlation between active commuting and cardiorespiratory fitness in children and adolescents (Muntaner-Mas et al., 2018). In contrast, our current investigation contradicts previous findings. Furthermore, our study indicates a lack of association between active commuting and depression and anxiety. We considered various factors to explain why active commuting might not be linked to depression and anxiety, such as the relatively lower intensity of active commuting (e.g., walking) in children and adolescents, potential biases in self-reported measures of depression and anxiety, and the lack of robust validation for assessing active commuting. This observation might also elucidate the absence of a significant association between MSE and the co-occurrence of depression and anxiety in students. For instance, even though students participate in active commuting between school and home and incorporate muscle-strengthening or toning exercises, the duration and intensity of these activities may be limited, potentially falling below the threshold required to alleviate depression and anxiety in adolescents. Due to the scarcity of comparable evidence in this study, additional observational and intervention studies could be warranted to investigate and validate the impact of active commuting on depression and anxiety among children and adolescents.

### 4.1 Practical implications

The strategy that optimizes the impact of sports on kids’ psychological health should be explored in future studies. Apart from that, it is essential to examine more game-based interventional and longitudinal research to enhance children’s self-awareness, inner motivation, and well-being, as well as mental and physiological results (Yan et al., 2023). Notably, the SAAFE (Supportive, Active, Autonomous, Fair, Enjoyable) principle provides a framework to design and deliver exercise sessions (Lubans et al., 2017), which was guided by the self-determination theory, these principles are applied to two HIIT researches (Hancox et al., 2018). To a certain extent, HIIT (high-intensity interval training) has an aversive nature. Still, if the design of dynamic physical activities can satisfy basic mental needs, it may be more interesting for children (Hancox et al., 2018). For example, participants can be offered choices of exercises and rest interval durations to satisfy autonomy and be given positive feedback to strengthen competence perception. Apart from that, performing vigorous physical activities in groups will become more enjoyable and meet the perception of relatedness. In this research, self-reported measures are adopted to gather all variables’ data, which can be influenced by participants’ social desirability and recall bias. Through cross-sectional design, the causal relationship may not be explained satisfactorily. Moreover, this study has some limitations. It should be noted that the data collection was conducted in 2021, which may result in differences between the findings and those of non-covid-19 pandemics. However, one previous study suggested a similar association between physical activity and muscle-strengthening exercises and mental health in adolescents before and during the COVID-19 pandemic (Burns et al., 2023). This emphasised the importance of physical activity in preventing depression and anxiety in adolescents, particularly in the period of the Covid-19 pandemic. Namely, except for the frequency type, no distinction is made by the movement type, and data support on the differentiation of different sports categories is not provided. Besides, during the COVID-19 pandemic, children and adolescents in China experienced heightened levels of anxiety and depression, particularly during periods of lockdowns and restrictions. Studies have shown increased psychological distress among youth attributed to factors such as social isolation, disrupted routines, and concerns about health and safety (Shanahan et al., 2022). These challenges were exacerbated by limitations on movement and social activities, which heightened feelings of loneliness and stress (Holmes et al., 2020). It is essential to conduct a prospective research design to gain a better understanding of the causal relations between sports participation, muscle-strengthening exercise, and active commuting with depression and anxiety and improve the success rate of intervention. In addition, the assessment of depression and anxiety was conducted via self-reported questionnaires. However, the potential for measurement bias may be introduced by participants’ prior experiences with similar scales. Finally, convenience sampling has a disadvantage. Specifically, it is likely not very objective because it may not represent the population. Given the limitations, conducting future research to resolve problems and gain more decisive evidence is essential.

## 5 Conclusion

The present study substantiated that sports participation was more effective in alleviating anxiety and depression in adolescents compared to AC and MSE. Future research should delve into strategies that enhance the mental health benefits of sports for youth. Optimising the effect size may also be achieved by focusing on neurobiological, behavioral, and psychosocial mechanisms.

## Data Availability

The raw data supporting the conclusions of this article will be made available by the authors, without undue reservation.
